# SCADET: A detection framework for AI-generated artwork integrating dynamic frequency attention and contrastive spectral analysis

**DOI:** 10.1371/journal.pone.0336328

**Published:** 2025-11-26

**Authors:** Xiaolong Zhang, Zekai Yu, Jianqiao Zhao

**Affiliations:** 1 School of Art and Design, Xihua University, Chengdu, China; 2 School of Computer Science and Technology, Hangzhou Dianzi University, Hangzhou, China; 3 Faculty of Fine and Applied Arts and Cultural Science, Mahasarakham University, Maha Sarakham, Thailand; West Virginia State University, UNITED STATES OF AMERICA

## Abstract

With the rapid development of generative AI technology, AI-generated images pose significant challenges for authenticity verification and originality validation. This paper proposes SCADET, a novel detection framework that integrates Dynamic Frequency Attention Network (DFAN) and Contrastive Spectral Analysis Network (CSAN). DFAN adaptively analyzes image frequency domain features and dynamically adjusts attention for different artistic styles, while CSAN establishes discriminative feature spaces through contrastive learning to enhance cross-model generalization capabilities. Comprehensive experiments on the AI-ArtBench dataset demonstrate that SCADET achieves AUC values of 0.962 and 0.801 in full image and local image detection tasks respectively, representing substantial improvements of 30.5% and 34.4% over baseline methods. Cross-model evaluation shows that the framework maintains stable performance across various generation techniques, with an average accuracy of 0.81 and low variance. Ablation studies validate the effectiveness of both DFAN and CSAN components. These results advance the field of AI-generated content detection and provide valuable insights for addressing authenticity challenges in digital media applications.

## 1 Introduction

### 1.1 Background

In recent years, generative AI technology has achieved breakthrough progress, especially with the rapid development of Generative Adversarial Networks (GANs), Diffusion Models, and Transformer-based text-to-image models (such as DALL-E, Midjourney, and Stable Diffusion), which have brought AI-generated images to unprecedented levels of quality and realism [1]. These technologies generate realistic visual content from text descriptions or reference images. They demonstrate enormous potential in creative industries, entertainment media, and digital art. However, as these technological capabilities improve, the boundary between AI-generated content and human creation is increasingly blurred, bringing unprecedented challenges and ethical dilemmas to multiple domains [2].

While this technological advancement brings innovative possibilities, it has also led to a series of negative social impacts [3]. In the art market, AI-generated works are being sold as human artists’ creations. This practice disrupts the value assessment system for artworks and undermines the protection of creators’ rights [4]. In the design industry, some practitioners use AI tools to quickly generate content and claim it as original design, undermining fair competition and professional value recognition in the industry; in the copyright domain, AI models generate works by learning specific artists’ styles and use them for commercial purposes without authorization, triggering complex intellectual property disputes [5]. In educational assessment, teachers struggle to distinguish between students’ original works and AI-assisted creations, affecting fair evaluation and accurate measurement of educational effectiveness. These issues not only damage creators’ legitimate rights but also threaten public trust in digital content [6].

Existing AI-generated image detection technologies face enormous challenges in keeping pace with the rapid evolution of generative models [7]. Traditional detection methods based on pixel-level features or simple statistical properties perform poorly when facing high-quality AI-generated images; deep learning-based detectors often perform well on specific datasets but have limited generalization ability, unable to effectively identify content created by new generative models [8]. More critically, as generative technologies continuously improve, newly generated images are increasingly difficult to distinguish from real photographs, making detection more challenging [9]. This “arms race” style of technological confrontation is intensifying, and without sufficiently powerful and adaptive detection technologies, society will struggle to address the risks brought by the misuse of AI-generated content .

Facing these challenges, developing efficient and robust AI-generated image detection technology has become a critical issue that urgently needs to be solved. This research aims to construct a detection framework that can adapt to various generative models and possess good generalization capabilities by combining innovative methods of frequency domain analysis and contrastive learning, providing technical support for maintaining integrity in creative industries, protecting creators’ rights, supporting fair assessment, and enhancing public trust. In an era where AI-generated content is increasingly prevalent, establishing such a “digital content authenticity defense line” not only has technical significance but also has profound social value, serving as an important guarantee for the healthy development of the digital ecosystem.

### 1.2 Literature review

In recent years, AI-assisted creativity as a revolutionary creative paradigm has triggered profound changes in the fields of art, design, and media content production [10,11]. The rapid development of generative AI technology has evolved from early basic image synthesis to today’s diffusion models and multimodal large language models capable of creating complex artworks. Research by Zhou and Lee [10] shows that text-to-image generative AI (such as Midjourney, Stable Diffusion, DALL-E) significantly enhances human creative productivity by 25%. These tools also improve the possibility of works gaining attention by 50%. These technologies are reshaping the workflow and value chain of creative industries: Kaljun and Kaljun [12] found that integrating generative AI tools in the conceptual phase of sustainable product design not only accelerates the ideation process but also promotes innovative solutions that meet environmental goals; Evangelidis et al. [13] emphasize the transformative role of AI in enhancing creativity and collaborative processes in education, proposing an ecosystem that integrates AI-assisted co-creation tools, story development, and digital exhibition boards, providing a comprehensive framework for revolutionizing art education. Nevertheless, this technological wave also brings unprecedented challenges. Agarwal [14] points out that AI is not the end of human creativity, but a powerful complementary tool; collaborating with AI can automate routine tasks and gain insights, leaving more time and energy for creative problem-solving. Molla [11] explores the challenges and controversies faced by AI-generated art, including issues of authorship, ownership, and the role of human imagination. These studies collectively highlight the urgency of developing reliable AI-generated content detection technologies, especially their critical role in maintaining integrity in the creative field and protecting creators’ rights.

The rise of AI-generated art has brought significant impact to traditional art markets, triggering reconsideration of artwork authentication and value assessment. Gjorgjieski [15] explores AI’s influence on various art forms including painting, sculpture, photography, and illustration, with special attention to the challenges faced by the art market and industry in terms of authorship, originality, and creative innovation. This view resonates with Zou’s research [16], which examines the development and impact of AI-generated content in contemporary painting, pointing out that copyright issues, market acceptance, and legal frameworks are the main obstacles to integrating AI art into traditional art fields. From an educational perspective, Zhou [17] studied the application of AI art generators in pre-service art teacher training, finding that although AI tools enhance artistic creativity and efficiency, their market impact in educational and commercial fields still needs careful evaluation. Kaushal and Mishra [18] researched the strategic impact of AI on creative industries, emphasizing AI’s role in shaping customer experiences and optimizing creative processes, while raising ethical concerns surrounding automated art creation. This balance between ethics and business is also discussed in Barat and Gulati’s research [19], which focuses on the application of AI-generated art in digital branding, analyzing how AI tools influence consumer behavior and the prospects of AI-driven art content in marketing through cases like Amazon and Netflix. Although Ahmadirad’s research [20] focuses on financial markets, its exploration of the boundary between AI-driven real market growth and speculative hype provides an important reference for understanding the value assessment of AI-generated art in art markets. These studies collectively indicate that as AI-generated art becomes more prevalent, there is an increasingly urgent need in the art market for effective technical tools to distinguish between human creations and AI-generated content, providing objective basis for artwork valuation, copyright protection, and market regulation.

The development of AI-generated content detection has evolved alongside advances in generation technologies. Early detection methods relied on identifying visual artifacts and statistical inconsistencies using traditional computer vision techniques [21]. With the rise of deep learning, CNN-based approaches became dominant, learning discriminative features to distinguish real from synthetic content [22]. Recent research has explored various enhancement strategies, including attention mechanisms to focus on important image regions and contrastive learning to improve detection robustness. However, most attention-based methods concentrate on spatial features, while contrastive approaches typically operate on standard image representations [23]. The combination of attention mechanisms with frequency analysis and the application of contrastive learning to alternative feature spaces remain less explored areas in current detection research.

To systematically evaluate the research status and limitations of existing AI-generated content detection methods, Table 1 provides a comparative analysis of recent representative studies. As can be seen from the table, although existing methods have achieved certain success under specific conditions, there are still obvious deficiencies in dealing with diverse artistic styles, local area detection, and cross-model generalization, which are precisely the core issues that the SCADET framework proposed in this research aims to solve.

**Table 1:** Comparative analysis of AI-generated content detection research.

Authors	Application Scenario	Research Content	Potential Limitations
Xiao & Zhao [24]	Detection of AI-generated art in different styles	Study of how input image type and style affect the accuracy of AI detection models, finding that AI classifiers perform better at detecting realistic-style AI-generated images	Performs poorly on traditional or animated styles, lacks adaptive analysis capabilities for different artistic styles, unable to dynamically adjust attention to different frequency band features
Ha et al. [25]	Distinguishing AI-generated art from human art	Evaluation of various AI-generated art detection techniques, comparing automated tools and human assessors, finding that expert artists and AI classifiers complement each other, with hybrid systems achieving the highest accuracy	Relies on human expert participation, difficult to achieve fully automated detection, lacks systematic frequency domain-based analysis methods, difficult to apply to large-scale art market regulation
Chinta et al. [26]	Classification of AI-generated art versus human art	Evaluation of CNN, VGG19, and ResNet50 models’ ability to distinguish AI-generated images from human-created artworks, with CNN model achieving the highest accuracy of 92.69%	Based on fixed model architecture and static feature extraction, lacks frequency domain analysis and contrastive learning capabilities, faces cross-model generalization problems, difficult to adapt to new generation technologies
Li et al. [27]	Adversarial AI-generated art detection	Introduction of the ARIA large-scale dataset containing over 140,000 images of different categories, benchmarking AI detectors, revealing challenges in adversarial settings	Detection performance significantly decreases in adversarial settings, lacks dynamic feature extraction capabilities, struggles with post-processed and hybrid content, lacks local region detection capabilities
Khan et al. [28]	Neural representation analysis of AI versus human art	Using deep learning to analyze neural representation patterns of AI-generated art and human-created artworks, finding that AI-generated art is close to modern abstract styles but lacks emotional complexity	Primarily focuses on art style analysis rather than detection methods, lacks algorithm design specifically for authenticity verification, cannot effectively process local image features, not suitable for actual authentication scenarios
Sanghvi et al. [29]	Research on design features of AI-generated art	Exploration of design features of AI-generated art in painting and animation, emphasizing differences between AI and human creation, discussing applications of deep learning techniques in detecting AI-generated artworks	Lacks quantitative detection methods and specific algorithm frameworks, does not address cross-model generalization issues, does not explore practical effectiveness in different application scenarios (such as art markets, educational assessment)
Gawali et al. [30]	Detection of AI-generated fake media	Review of deep learning methods for detecting AI-generated fake media, including neural network-based classifiers, emphasizing the need for continuous improvement of detection methods as generation technologies develop	Primarily a review, lacks innovative detection frameworks, does not propose methods for dynamically adapting to new generation technologies, no specialized design for frequency domain features and contrastive learning
Fraser et al. [31]	Evaluation of AI-generated text and image detection methods	Evaluation of state-of-the-art AI detection methods, discussing watermarks, statistical analysis, and machine learning classifiers, providing insights on the effectiveness of current technologies	Does not fully address frequency domain feature analysis issues, lacks specialized design for local region detection, does not propose effective cross-model generalization strategies, limited adaptability to new generation technologies

In summary, although existing AI-generated image detection research has made certain progress, it still faces three core challenges. Most methods use static feature extraction strategies and lack adaptive analysis capabilities for different artistic styles, as reflected in Table 1. Existing methods perform poorly in cross-model generalization, struggling to cope with rapidly iterating generation technologies. Additionally, there are significant deficiencies in local region detection and anti-interference capabilities, limiting effectiveness in practical application scenarios. These limitations directly hinder the widespread application of AI-generated content detection technology in art markets, design industries, and educational assessment. These limitations directly hinder the widespread application of AI-generated content detection technology in art markets, design industries, and educational assessment. Building on existing attention mechanism research and contrastive learning approaches, the SCADET framework extends these concepts through two main innovations. While previous attention methods focus on spatial image features, SCADET applies dynamic attention to frequency domain features, allowing adaptive analysis of different artistic styles. Similarly, rather than using contrastive learning on conventional image features, SCADET employs contrastive analysis on frequency-based representations to enhance cross-model detection capabilities. This combination of frequency-adaptive attention with contrastive feature mapping addresses the identified limitations while providing enhanced detection performance across various artistic styles and generation technologies.

### 1.3 Our contributions

- We propose the SCADET framework, innovatively combining Dynamic Frequency Attention Network (DFAN) and Contrastive Spectral Analysis Network (CSAN). DFAN, through an adaptive frequency band weight allocation mechanism, can dynamically adjust the importance of frequency domain features according to different artistic styles, effectively capturing overly regular textures and unnatural details in AI-generated images; CSAN utilizes contrastive learning to establish feature difference mapping between AI-generated and human-created images, significantly enhancing the detection system’s discriminative ability and cross-model generalization. The synergistic effect of these two mechanisms enables SCADET to effectively cope with the rapid iteration of generation technologies while maintaining high detection accuracy.
- We construct a comprehensive experimental evaluation framework, validating SCADET’s effectiveness across multiple application scenarios and generation technologies. Full image detection experiments demonstrate the algorithm’s superior performance under standard conditions (AUC=0.962); local image detection experiments verify its robustness in situations where only partial features are visible (AUC=0.801); cross-model experiments quantify its stable performance across five different generation technologies (average accuracy 0.81, standard deviation 0.026). Ablation studies systematically analyze the contributions of DFAN and CSAN, as well as the detectability characteristics of different AI generation technologies, providing scientific basis for theoretical research on detection algorithms.
- We establish a practical AI-generated content detection solution targeting the actual needs in art markets, design industries, and educational assessment. Research findings show that under low false positive rate conditions (FPR=0.05), SCADET can still maintain a high detection rate (TPR=0.89), suitable for high-value scenarios such as artwork authentication; it performs excellently when handling unseen generation models (accuracy 0.76), capable of addressing technological iterations; it remains effective under compressed images (retention rate 0.93) and low-resolution conditions (recognition rate 0.88), meeting diverse requirements of practical applications. These characteristics make SCADET a powerful tool for maintaining integrity in creative industries, protecting original rights, and supporting fair assessment.

In summary, this research addresses the challenges in AI-generated artwork detection by proposing the SCADET framework, which combines dynamic frequency analysis with contrastive learning mechanisms. The framework aims to improve adaptive style analysis, cross-model generalization, and local region detection capabilities compared to existing methods. Experimental validation shows its effectiveness across different generation technologies and application scenarios, providing a practical approach for authenticity verification in creative industries, intellectual property protection, and educational assessment.

## 2 Our approach

### 2.1 Problem statement

To precisely describe the AI-generated image detection problem, it is necessary to construct a rigorous mathematical model. Let 𝒳 represent the image space, ℛ⊂𝒳 represent the distribution of real images, and 𝒢⊂𝒳 represent the distribution of AI-generated images. There are inherent differences in distributional characteristics between real images and AI-generated images, which can be quantified through distribution divergence:

$$D(ℛ\|𝒢)=\int_{𝒳}p_{ℛ}(x)\log\frac{p_{ℛ}(x)}{p_{𝒢}(x)}dx$$ (1)

where D(ℛ‖𝒢) represents the KL divergence between the distributions of the two classes of images, and $p_{ℛ}(x)$ and $p_{𝒢}(x)$ represent the probability density functions of real images and AI-generated images, respectively. However, with the advancement of generation technologies such as Midjourney and DALL-E, this distribution difference (D(ℛ‖𝒢)) is gradually decreasing, making it increasingly difficult to distinguish between authentic and fake works in design portfolios and art markets. Formalizing AI-generated image detection as a binary classification problem, the objective function can be expressed as:

$$\min_{\theta }{𝔼}_{x\sim ℛ}[ℒ(f_{\theta }(x),0)]+{𝔼}_{x\sim 𝒢}[ℒ(f_{\theta }(x),1)]$$ (2)

where $f_{\theta }:𝒳\to [0,1]$ is a detector with parameters *θ*, and ℒ is a loss function (such as cross-entropy loss). In-depth research indicates that AI-generated images exhibit unique characteristics in the frequency domain, which can be precisely analyzed through Fourier transform:

$$F(u,v)=\sum_{x=0}^{M-1}\sum_{y=0}^{N-1}I(x,y)e^{-j2\pi (\frac{ux}{M}+\frac{vy}{N})}$$ (3)

where *I*(*x*,*y*) is the pixel value of the image in the spatial domain, and *F*(*u*,*v*) is its representation in the frequency domain. This transformation reveals key features of AI-generated images—they exhibit statistical properties different from human-created works in specific regions of the spectrum (such as high-frequency parts). One of the main challenges faced by current detection technologies is limited generalization ability to new generation models, which can be quantitatively represented by the generalization gap:

$$\Delta (f,{𝒢}_{\text{train}},{𝒢}_{\text{test}})=|{𝔼}_{x\sim {𝒢}_{\text{train}}}[f(x)=1]-{𝔼}_{x\sim {𝒢}_{\text{test}}}[f(x)=1]|$$ (4)

where ${𝒢}_{\text{train}}$ and ${𝒢}_{\text{test}}$ represent the distributions of AI-generated images in the training and test sets, respectively, and *Δ* represents the performance gap of the detector on these two distributions. A larger *Δ* value means the detection system struggles to identify content created by unseen generation models, which greatly limits its practicality in the rapidly evolving environment of AI artistic creation and its ability to effectively protect the original rights of artists and designers. Different types of AI generation technologies (such as GANs, diffusion models, multimodal large language models, etc.) produce images with significant differences in features, which can be quantified using Wasserstein distance:

$$d({𝒢}_{i},{𝒢}_{j})=\|\mu_{i}-\mu_{j}{\|}_{2}+\text{tr}(\Sigma_{i}+\Sigma_{j}-2(\Sigma_{i}\Sigma_{j}{)}^{1/2})$$ (5)

where ${𝒢}_{i}$ and ${𝒢}_{j}$ represent distributions of different generation models, and $\mu_{i}$, $\mu_{j}$ and $\Sigma_{i}$, $\Sigma_{j}$ are their mean vectors and covariance matrices, respectively. The ideal detection system should possess both high accuracy and excellent generalization ability, which can be formulated as an optimization problem:

$$f^{*}=\arg\min_{f\in ℱ}\left\{{𝔼}_{x\sim ℛ}[ℒ(f(x),0)]+{𝔼}_{x\sim \cup_{i}{𝒢}_{i}}[ℒ(f(x),1)]+\lambda \Omega (f)\right\}$$ (6)

where ℱ represents the space of all possible detectors, $\cup_{i}{𝒢}_{i}$ represents the union of various AI generation model distributions, Ω(f) is a regularization term, and *λ* is a balancing parameter. Combining the above analyses, the core problem of AI-generated image detection can be formalized as Problem 1.

**Problem 1.** *Given the image space* 𝒳 *and the distributions of real images* ℛ *and AI-generated images* $𝒢=\cup_{i}{𝒢}_{i}$ *(including various generation models) within it, how to design a detector* f:𝒳→[0,1] *such that it satisfies:*

$$\min_{f}\left\{{𝔼}_{x\sim ℛ}[ℒ(f(x),0)]+{𝔼}_{x\sim 𝒢}[ℒ(f(x),1)]\right\}$$ (7)

$$\text{s.t.}\max_{i}\Delta (f,{𝒢}_{\text{train}},{𝒢}_{i})<\epsilon$$ (8)

*where ε is the acceptable upper limit of generalization error.*

Problem 1 establishes the mathematical framework for AI-generated image detection, where the detector *f* needs to minimize classification error while ensuring good generalization to unseen generation models. Eq (7) formalizes the classification objective, requiring the detector to accurately distinguish between human creations (ℛ) and AI-generated content (𝒢); the constraint condition (8) ensures that the generalization gap of the detector from the training distribution to any target distribution does not exceed the threshold *ε*.

### 2.2 DFAN: Capturing unnatural details in images

#### 2.2.1 Comparative analysis of DFAN and traditional algorithms in unnatural detail capture capability.

- Existing AI-generated image detection methods employ static frequency domain filtering strategies, lacking necessary adaptive mechanisms [32]. Such methods assign fixed weights to all frequency band regions, making it difficult to distinguish between artistic style variations and AI-generated spectral anomalies [33]. Performing particularly poorly when processing works rich in high-frequency details. Due to the lack of dynamic adjustment capabilities in the feature extraction process, traditional algorithms experience significantly decreased generalization performance when facing generation models outside the training distribution, failing to effectively address rapidly evolving AI generation technologies [34].
- The DFAN algorithm achieves efficient capture of unnatural details through a dynamic frequency attention mechanism. This algorithm adaptively adjusts frequency band attention according to input image characteristics. It automatically optimizes detection strategies for spectral distributions of different artistic styles. This adaptive characteristic enables DFAN to precisely distinguish between reasonable spectral variations and AI-generated artifacts in complex artistic styles, with significantly better capture capability for high-frequency detail regions than traditional methods.

**Fig 1:**
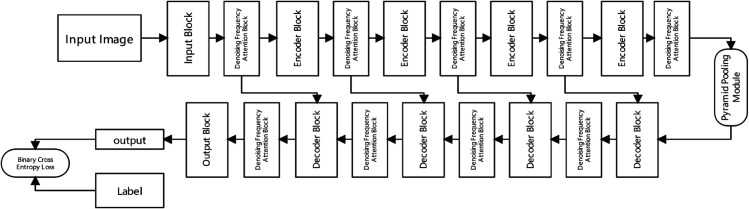
Dynamic frequency-adaptive image authenticator.

Fig 1 illustrates the proposed Dynamic Frequency Attention Network (DFAN) architecture for AI-generated image detection. Unlike traditional methods that employ static frequency domain filtering with fixed weights for all frequency bands, DFAN introduces an adaptive mechanism through dynamic frequency attention. This novel approach enables the network to precisely distinguish between legitimate artistic style variations and AI-generated spectral anomalies.

#### 2.2.2 DFAN’s deep feature-based unnatural region recognition mechanism.

The Dynamic Frequency Attention Network (DFAN) focuses on identifying unnatural feature regions in AI-generated artworks and design works. Given a suspicious artwork $I\in {\mathbb{R}}^{H\times W\times 3}$, DFAN first maps it to a discriminative information-rich spectral space through an enhanced multi-scale frequency domain transformation:

$$F(u,v)=\sum_{x=0}^{H-1}\sum_{y=0}^{W-1}[I(x,y)\cdot w(x,y)\cdot e^{-j2\pi \left(\frac{ux}{H}+\frac{vy}{W}\right)}\cdot \;\left(1+\alpha \cdot \sqrt{u^{2}+v^{2}}+\beta \cdot \exp\left(-\frac{(u-\frac{H}{2}{)}^{2}+(v-\frac{W}{2}{)}^{2}}{2\sigma^{2}}\right)\right)\cdot \;\left(1+\gamma \cdot \frac{{\text{var}}_{x^{\prime },y^{\prime }}(I(x^{\prime },y^{\prime }))}{\max_{x^{\prime },y^{\prime }}{\text{var}}_{x^{\prime \prime },y^{\prime \prime }}(I(x^{\prime \prime },y^{\prime \prime }))}\right)]$$ (9)

where *w*(*x*,*y*) is a window function used to reduce spectral leakage, (*u*,*v*) are frequency domain coordinates, *α* controls the degree of high-frequency enhancement, *β* controls the degree of low-frequency enhancement, *σ* is a Gaussian decay parameter, and *γ* is a local variability weight. DFAN employs adaptive frequency band filters to extract discriminative features from the enhanced spectrum. The filter response function is jointly determined by frequency domain position, directional selectivity, and energy distribution:

$$M_{k}(u,v)=\exp\left(-\frac{(r(u,v)-r_{k}{)}^{2}}{2\sigma_{r}^{2}}\right)\cdot \left(1+\delta_{k}\cdot \cos^{2}(\theta (u,v)-\theta_{k})\right)\cdot \;\left(1+\sum_{i=1}^{N}\eta_{k,i}\cdot \sin^{2}\left(\frac{\pi \cdot i\cdot r(u,v)}{r_{\max}}\right)+\sum_{j=1}^{M}\zeta_{k,j}\cdot \cos\left(j\cdot (\theta (u,v)-\phi_{k})\right)\right)\cdot \;\frac{1}{1+\exp\left(-\lambda_{k}\cdot \left(|F(u,v){|}^{2}-\tau_{k}\right)\right)}\cdot \left(1+\kappa_{k}\cdot \frac{{\text{var}}_{u^{\prime },v^{\prime }}(|F(u^{\prime },v^{\prime })|)}{(1+|u-\frac{H}{2}|{)}^{\rho_{1}}\cdot (1+|v-\frac{W}{2}|{)}^{\rho_{2}}}\right)$$ (10)

where $r(u,v)=\sqrt{(u-\frac{H}{2}{)}^{2}+(v-\frac{W}{2}{)}^{2}}$ is the radial distance, $\theta (u,v)=\tan^{-1}(\frac{v-\frac{W}{2}}{u-\frac{H}{2}})$ is the angle, *r*_*k*_ and $\theta_{k}$ are the center frequency and direction of the *k*-th filter, $\delta_{k}$ controls directional selectivity, $\eta_{k,i}$ and $\zeta_{k,j}$ are radial and angular modulation coefficients, $\lambda_{k}$ and $\tau_{k}$ control the energy adaptive threshold, and $\kappa_{k}$, $\rho_{1}$, and $\rho_{2}$ regulate local variability response. For each frequency band, DFAN constructs deep feature representations, capturing complex statistical anomalies in AI-generated artworks:

$${𝐳}_{k}=\;{\text{CNN}}_{k}\left(|F|⊙M_{k}\right)+{\text{Phase}}_{k}\left(∠F⊙M_{k}\right)+\;\mu_{k}\cdot {\text{HighOrder}}_{k}\left(|F|⊙M_{k},(|F|⊙M_{k}{)}^{2},(|F|⊙M_{k}{)}^{3}\right)+\;\nu_{k}\cdot {\text{CrossSpectral}}_{k}\left(|F|⊙M_{k},∠F⊙M_{k},\text{Re}(F)⊙M_{k},\text{Im}(F)⊙M_{k}\right)+\;\xi_{k}\cdot {\text{MultiScale}}_{k}\left(\{|F_{s_{l}}|⊙M_{k,s_{l}}{\}}_{l=1}^{L}\right)+\omega_{k}\cdot {\text{Contextual}}_{k}\left(I,|F|⊙M_{k},\{{\text{Grad}}^{n}(I){\}}_{n=1}^{N_{g}}\right)$$ (11)

where ${\text{CNN}}_{k}$, ${\text{Phase}}_{k}$, ${\text{HighOrder}}_{k}$, ${\text{CrossSpectral}}_{k}$, ${\text{MultiScale}}_{k}$, and ${\text{Contextual}}_{k}$ extract amplitude features, phase features, high-order moment features, amplitude-phase interaction features, multi-scale features, and contextual features respectively, ∠F represents the phase, $F_{s_{l}}$ is the spectrum at scale *s*_*l*_, ${\text{Grad}}^{n}(I)$ is the *n*-th order gradient of the image, and $\mu_{k}$, $\nu_{k}$, $\xi_{k}$, and $\omega_{k}$ are feature fusion weights. The core of DFAN is the dynamic frequency attention mechanism, focusing on the most discriminative frequency bands through complex adaptive weight calculation:

$$\alpha_{k}=\frac{\exp\left(W_{a}^{T}\cdot {𝐳}_{k}+b_{a}+\sum_{j=1}^{K}\beta_{k,j}\cdot W_{a,j}^{T}\cdot {𝐳}_{j}\right)}{\sum_{i=1}^{K}\exp\left(W_{a}^{T}\cdot {𝐳}_{i}+b_{a}+\sum_{j=1}^{K}\beta_{i,j}\cdot W_{a,j}^{T}\cdot {𝐳}_{j}\right)}\cdot \;\left(1+\gamma_{1}\cdot \frac{\|{𝐳}_{k}{\|}_{2}^{2}}{\frac{1}{K}\sum_{j=1}^{K}\|{𝐳}_{j}{\|}_{2}^{2}}+\gamma_{2}\cdot \frac{\text{entropy}({𝐳}_{k})}{\max_{j}\text{entropy}({𝐳}_{j})}\right)\cdot \;\left(1+\gamma_{3}\cdot \frac{D_{KL}({𝐳}_{k}\|{\bar{𝐳}}_{ℛ,k})}{\frac{1}{K}\sum_{j=1}^{K}D_{KL}({𝐳}_{j}\|{\bar{𝐳}}_{ℛ,j})}+\gamma_{4}\cdot \frac{\exp\left(-\frac{(r_{k}-r_{\text{opt}}{)}^{2}}{2\sigma_{r}^{2}}\right)}{\frac{1}{K}\sum_{j=1}^{K}\exp\left(-\frac{(r_{j}-r_{\text{opt}}{)}^{2}}{2\sigma_{r}^{2}}\right)}\right)$$ (12)

where *W*_*a*_, *b*_*a*_, and *W*_*a*,*j*_ are attention network parameters, $\beta_{k,j}$ are frequency band interaction coefficients, $\gamma_{1}$ through $\gamma_{4}$ control the influence of different attention modulation factors, entropy(·) calculates feature entropy, *D*_*KL*_ is the KL divergence, ${\bar{𝐳}}_{ℛ,k}$ is the average feature of real artworks in the *k*-th frequency band, $r_{\text{opt}}$ is the empirical optimal frequency, *r*_*k*_ is the center frequency of the *k*-th frequency band, and $\sigma_{r}$ is the frequency distance scaling factor. Through dynamic attention weights, DFAN constructs comprehensive frequency domain feature representations, enhancing the detection capability for AI-generated artworks:

$${𝐳}_{\text{enh}}=\sum_{k=1}^{K}\alpha_{k}\cdot {𝐳}_{k}+\sum_{k=1}^{K}\sum_{j=k+1}^{K}\alpha_{k}\alpha_{j}\cdot g({𝐳}_{k},{𝐳}_{j})+\sum_{k=1}^{K}\sum_{j=k+1}^{K}\sum_{l=j+1}^{K}\alpha_{k}\alpha_{j}\alpha_{l}\cdot h({𝐳}_{k},{𝐳}_{j},{𝐳}_{l})+\;\lambda_{1}\cdot \sum_{k=1}^{K}\alpha_{k}^{2}\cdot f_{1}({𝐳}_{k},{𝐳}_{k})+\lambda_{2}\cdot \sum_{k=1}^{K}\alpha_{k}^{3}\cdot f_{2}({𝐳}_{k},{𝐳}_{k},{𝐳}_{k})+\;\lambda_{3}\cdot \left(\sum_{k=1}^{K}\alpha_{k}\cdot {𝐳}_{k}\right)⊙\sigma \left(W_{1}\cdot \sum_{k=1}^{K}\alpha_{k}\cdot {𝐳}_{k}+b_{1}\right)+\;\lambda_{4}\cdot \text{Attention}\left(\sum_{k=1}^{K}\alpha_{k}\cdot {𝐳}_{k},\{{𝐳}_{k}{\}}_{k=1}^{K},\{{𝐳}_{k}{\}}_{k=1}^{K}\right)$$ (13)

where g(·,·) and h(·,·,·) are second-order and third-order feature interaction functions respectively, *f*_1_ and *f*_2_ calculate second-order and third-order self-interaction features, *W*_1_ and *b*_1_ are non-linear transformation parameters, *σ* is the sigmoid activation function, Attention(·,·,·) is a self-attention mechanism, and $\lambda_{1}$ through $\lambda_{4}$ are feature fusion coefficients. This high-order feature fusion can capture subtle statistical anomalies in AI-generated artworks, providing crucial evidence for authenticity verification. DFAN ultimately constructs a pixel-level anomaly map, quantifying the degree of unnaturalness at each position in the image:

$$A_{\text{map}}(i,j)=\sum_{k=1}^{K}\alpha_{k}\cdot \frac{{\left\|\phi_{k}(F_{i,j})-\mu_{ℛ,k}\right\|}_{2}^{2}}{\sigma_{ℛ,k}^{2}+\epsilon }\cdot \left(1+\eta_{1}\cdot \exp\left(-\frac{\|[i,j]-[i_{c},j_{c}]{\|}_{2}^{2}}{2\sigma_{c}^{2}}\right)\right)\cdot \;\left(1+\eta_{2}\cdot \frac{D_{KL}(p_{\phi_{k}(F_{i,j})}\|p_{\phi_{k}(F_{ℛ})})}{\max_{i^{\prime },j^{\prime }}D_{KL}(p_{\phi_{k}(F_{i^{\prime },j^{\prime }})}\|p_{\phi_{k}(F_{ℛ})})}\right)\cdot \;\left(1+\eta_{3}\cdot \frac{{\text{var}}_{\left(i^{\prime },j^{\prime })\in 𝒩(i,j\right)}(\phi_{k}(F_{i^{\prime },j^{\prime }}))}{\frac{1}{HW}\sum_{i^{\prime }=1}^{H}\sum_{j^{\prime }=1}^{W}{\text{var}}_{\left(i^{\prime \prime },j^{\prime \prime })\in 𝒩(i^{\prime },j^{\prime }\right)}(\phi_{k}(F_{i^{\prime \prime },j^{\prime \prime }}))}\right)$$ (14)

where *F*_*i*,*j*_ represents the local frequency domain features centered at position (*i*,*j*), $\phi_{k}$ is the feature mapping function for the *k*-th frequency band, $\mu_{ℛ,k}$ and $\sigma_{ℛ,k}^{2}$ are the mean and variance of the corresponding features of real artworks, *ε* is a numerical stability constant, $\left[i_{c},j_{c}\right]$ are the image center coordinates, $\sigma_{c}$ controls center preference decay, $p_{\phi_{k}(F_{i,j})}$ and $p_{\phi_{k}(F_{ℛ})}$ are the distributions of local features and reference features respectively, 𝒩(i,j) represents the neighborhood of point (*i*,*j*), and $\eta_{1}$ through $\eta_{3}$ are anomaly metric weights. This comprehensive anomaly map enables art experts to intuitively understand the distribution of AI generation traces, providing interpretable support for authentication results.

**Theorem 1. (Frequency domain art feature separability).** *For real artwork distribution* ℛ *and AI-generated artwork distribution* 𝒢, *there exists a frequency band decomposition* $\left\{B_{1},B_{2},\ldots ,B_{K}\right\}$ *and corresponding feature mappings* $\left\{\phi_{1},\phi_{2},\ldots ,\phi_{K}\right\}$, *such that the following inequality holds:*

$${𝔼}_{x\sim 𝒢}\left[\sum_{k=1}^{K}\omega_{k}\cdot D_{KL}(\phi_{k}(F_{x})\|\phi_{k}(F_{ℛ}))+\sum_{k=1}^{K}\sum_{j=k+1}^{K}\omega_{k,j}\cdot D_{JS}(\phi_{k}(F_{x})\|\phi_{j}(F_{x}))\right]>\;{𝔼}_{x,y\sim ℛ}\left[\sum_{k=1}^{K}\omega_{k}\cdot D_{KL}(\phi_{k}(F_{x})\|\phi_{k}(F_{y}))+\sum_{k=1}^{K}\sum_{j=k+1}^{K}\omega_{k,j}\cdot D_{JS}(\phi_{k}(F_{x})\|\phi_{j}(F_{x}))\right]+\delta$$ (15)

*where F*_*x*_ *represents the frequency domain representation of artwork x,* $F_{ℛ}$ *represents the frequency domain distribution of real artworks, D*_*KL*_ *is the KL divergence, D*_*JS*_ *is the Jensen-Shannon divergence,* $\omega_{k}$ *and* $\omega_{k,j}$ *are single-frequency band and cross-frequency band weights respectively, and* δ>0 *is the statistical significance threshold.*

Based on the above theorem, an important corollary regarding AI creation feature distribution can be derived:

**Corollary 1 (Structured locality of creation anomalies).** *For an AI-generated artwork* x~𝒢, *there exists a set of spatial regions* $\Omega_{x}\subset \{1,2,\ldots ,H\}\times \{1,2,\ldots ,W\}$, *satisfying the following conditions:*

$$\frac{1}{\left|\Omega_{x}\right|}\sum_{(i,j)\in \Omega_{x}}A_{map}(i,j)>\gamma \cdot \frac{1}{HW-|\Omega_{x}|}\sum_{(i,j)\notin \Omega_{x}}A_{map}(i,j)\;and\;\frac{\left|\Omega_{x}\right|}{HW}<\eta \;and\;\frac{1}{\left|\partial \Omega_{x}\right|}\sum_{(i,j)\in \partial \Omega_{x}}A_{map}(i,j)>\frac{1}{\left|\Omega_{x}⧵\partial \Omega_{x}\right|}\sum_{(i,j)\in \Omega_{x}⧵\partial \Omega_{x}}A_{map}(i,j)$$ (16)

*where* $\left|\Omega_{x}\right|$ *represents the region size,* γ>1 *is the contrast coefficient,* η∈(0,1) *is the area constraint,* $\partial \Omega_{x}$ *represents the region boundary, and* $A_{\text{map}}$ *is defined by* Eq (14).

### 2.3 CSAN: Image feature difference mapping

#### 2.3.1 Differences between CSAN and classical feature mapping methods.

- Classical feature mapping methods use static feature spaces and simple metric standards, which have inherent limitations when dealing with AI-generated image recognition [35]. These methods rely on predefined feature embeddings and simple distance metrics, making it difficult to capture the essential differences between real and AI-generated artworks [36]. Traditional methods lack effective modeling of intra-class diversity and inter-class similarity when constructing feature space decision boundaries. This limitation results in poor performance when facing highly realistic AI-generated content [37].
- CSAN reconstructs the feature difference mapping mechanism through a contrastive learning framework, achieving a key technological breakthrough. This algorithm dynamically constructs discriminative representations, creating clear separation between real artworks and AI-generated works in the feature space. Through the contrastive relationship of positive and negative sample pairs, CSAN reinforces inter-class differences while maintaining intra-class consistency, capable of capturing subtle statistical features that are difficult to distinguish in direct feature space. The feature invariance implicit regularization introduced by the contrastive learning framework significantly enhances the model’s generalization ability on new generation technologies, providing more reliable technical support for art authentication and design originality verification.

#### 2.3.2 CSAN image feature difference mapping mechanism.

The Contrastive Spectral Analysis Network (CSAN) constructs feature difference mapping between AI-generated images and human-created images through contrastive learning, providing an advanced method for essentially distinguishing authentic and fake artworks. CSAN first extracts discriminative representations rich in style features from artworks through complex spectral domain transformations. Given an artwork $I\in {\mathbb{R}}^{H\times W\times 3}$ to be authenticated, the spectral domain feature extraction process can be represented as:

$$S(I)=\;\Psi \left(ℱ(I),𝒲(I),DCT(I),WT(I)\right)\cdot \;\left(1+\alpha_{1}\cdot \frac{\|\nabla I{\|}_{F}^{2}}{\|\nabla {𝒢}_{\sigma }*I{\|}_{F}^{2}+\epsilon }\right)\cdot \left(1+\alpha_{2}\cdot \frac{\text{var}(\text{Lap}(I))}{\text{var}(I)+\epsilon }\right)\cdot \;\left(1+\alpha_{3}\cdot \frac{\sum_{i=1}^{n}\lambda_{i}(\text{Hess}(I))}{\text{tr}(\text{Hess}(I))+\epsilon }\right)\cdot \left(1+\alpha_{4}\cdot \frac{H(p_{I})}{H(p_{𝒰})}\right)\cdot \;\prod_{l=1}^{L}{\left(1+\alpha_{5,l}\cdot \frac{\|{ℱ}_{l}(I)-\mu_{{ℱ}_{l}(ℛ)}{\|}_{2}^{2}}{\sigma_{{ℱ}_{l}(ℛ)}^{2}+\epsilon }\right)}^{\beta_{l}}$$ (17)

where Ψ is the spectral domain feature fusion function, ℱ, 𝒲, DCT, and WT represent Fourier transform, wavelet transform, discrete cosine transform, and Walsh transform respectively, ∇I captures gradient features of brush strokes and lines, ${𝒢}_{\sigma }$ is a Gaussian smoothing kernel, Lap and Hess calculate texture details and shape features respectively, $\lambda_{i}(\text{Hess}(I))$ are the eigenvalues of shape curvature, *H*(*p*_*I*_) quantifies the complexity of color distribution, ${ℱ}_{l}$ analyzes artistic elements at different scales, $\mu_{{ℱ}_{l}(ℛ)}$ and $\sigma_{{ℱ}_{l}(ℛ)}^{2}$ represent reference features of real artworks, and $\alpha_{1}$ to $\alpha_{4}$, $\alpha_{5,l}$, and $\beta_{l}$ are feature weighting parameters. Given the spectral domain feature *S*(*I*), the encoder $E_{\theta }$ maps it to a discriminative feature space:

$$E_{\theta }(S(I))=\;{\text{CNN}}_{\theta }\left(S(I)\right)+\lambda_{1}\cdot {\text{Transformer}}_{\theta }\left(S(I)\right)+\;\lambda_{2}\cdot {\text{Residual}}_{\theta }\left(S(I),\{A_{\text{map}}^{\left(k\right)}{\}}_{k=1}^{K}\right)+\;\lambda_{3}\cdot {\text{NonLocal}}_{\theta }\left(S(I),\{S_{j}(I){\}}_{j=1}^{J}\right)\cdot \left(1+\gamma \cdot \frac{H(S(I))}{\max_{j}H(S_{j}(I))}\right)+\;\lambda_{4}\cdot {\text{Graph}}_{\theta }\left(\{S(I^{\left(r\right)}){\}}_{r=1}^{R},\{A^{\left(r,s\right)}{\}}_{r,s=1}^{R}\right)$$ (18)

where ${\text{CNN}}_{\theta }$ captures local brush stroke features, ${\text{Transformer}}_{\theta }$ analyzes overall compositional relationships, ${\text{Residual}}_{\theta }$ focuses on suspicious regions identified by DFAN, ${\text{NonLocal}}_{\theta }$ detects remote semantic consistency, ${\text{Graph}}_{\theta }$ establishes a network of associations between artistic elements, $\{A_{\text{map}}^{\left(k\right)}{\}}_{k=1}^{K}$ is the collection of anomaly maps detected by DFAN, $\{S_{j}(I){\}}_{j=1}^{J}$ are multi-scale features, $\{S(I^{\left(r\right)}){\}}_{r=1}^{R}$ are key region features (such as facial features in portraits, fabric textures, light-shadow transitions, etc.), $\{A^{\left(r,s\right)}{\}}_{r,s=1}^{R}$ is the relationship matrix between regions, $\lambda_{1}$ to $\lambda_{4}$ are network fusion weights, and *γ* is an entropy modulation coefficient. For the contrastive learning process, CSAN designs a feature projection mechanism to enhance the distinctiveness of authentic and fake artwork features:

$$z_{I}=P_{\phi }\left(E_{\theta }(S(I))\right)={\text{MLP}}_{\phi }\left(E_{\theta }(S(I))\right)\cdot \;\left(1+\alpha \cdot \frac{\|E_{\theta }(S(I))-\mu_{E_{\theta }(S(ℛ))}{\|}_{2}^{2}}{\sigma_{E_{\theta }(S(ℛ))}^{2}+\epsilon }\right)\cdot \;\left(1+\beta \cdot \frac{\sum_{k=1}^{K}\alpha_{k}\cdot \frac{1}{\left|A_{\text{map}}^{\left(k\right)}\right|}\sum_{i,j}A_{\text{map}}^{\left(k\right)}(i,j)\cdot E_{\theta }(S(I){)}_{i,j}}{\sum_{k=1}^{K}\alpha_{k}\cdot \frac{1}{\left|A_{\text{map}}^{\left(k\right)}\right|}\sum_{i,j}A_{\text{map}}^{\left(k\right)}(i,j)}\right)\cdot \;\left(1+\gamma \cdot \frac{1}{N_{\Omega }}\sum_{(i,j)\in \Omega_{I}}\frac{E_{\theta }(S(I){)}_{i,j}}{\frac{1}{N-N_{\Omega }}\sum_{(i,j)\notin \Omega_{I}}E_{\theta }(S(I){)}_{i,j}}\right)$$ (19)

where $P_{\phi }$ is the projection head, ${\text{MLP}}_{\phi }$ is a multilayer perceptron, $\mu_{E_{\theta }(S(ℛ))}$ and $\sigma_{E_{\theta }(S(ℛ))}^{2}$ represent the typical feature distribution of real artworks, $\alpha_{k}$ is the key frequency band weight determined by DFAN, $A_{\text{map}}^{\left(k\right)}$ is the anomaly map, $\Omega_{I}$ is the set of high anomaly regions (such as eyes, fingers, etc. in AI-generated paintings where problems frequently occur), $N_{\Omega }$ is the number of pixels in high anomaly regions, *N* is the total number of pixels, and *α*, *β*, and *γ* are feature enhancement coefficients. CSAN adopts a loss function based on multi-positive sample contrastive learning, a design that considers the reality of diverse artistic styles:

$${ℒ}_{\text{contrast}}=-\frac{1}{\left|ℬ\right|}\sum_{I\in ℬ}\log\frac{\sum_{I^{+}\in 𝒫(I)}\exp\left(\text{sim}(z_{I},z_{I^{+}})/\tau \right)\cdot w(I,I^{+})}{\sum_{I^{\prime }\in ℬ⧵\{I\}}\exp\left(\text{sim}(z_{I},z_{I^{\prime }})/\tau \right)\cdot w(I,I^{\prime })}-\;\lambda_{\text{struct}}\cdot \frac{1}{\left|{ℬ}_{ℛ}\right|}\sum_{I\in {ℬ}_{ℛ}}\frac{1}{\left|𝒫(I)\right|}\sum_{I^{+}\in 𝒫(I)}\log\frac{\exp\left(\text{sim}(z_{I},z_{I^{+}})/\tau_{s}\right)}{\sum_{I^{\prime }\in {ℬ}_{ℛ}⧵\{I\}}\exp\left(\text{sim}(z_{I},z_{I^{\prime }})/\tau_{s}\right)}-\;\lambda_{\text{adv}}\cdot \frac{1}{\left|{ℬ}_{𝒢}\right|}\sum_{I\in {ℬ}_{𝒢}}\log\left(1-\frac{\exp\left(\text{sim}(z_{I},z_{\text{proto}}^{𝒢})/\tau_{a}\right)}{\exp\left(\text{sim}(z_{I},z_{\text{proto}}^{𝒢})/\tau_{a}\right)+\exp\left(\text{sim}(z_{I},z_{\text{proto}}^{ℛ})/\tau_{a}\right)}\right)$$ (20)

where ℬ represents the training batch, ${ℬ}_{ℛ}$ and ${ℬ}_{𝒢}$ are batches of real and AI-generated artworks respectively, 𝒫(I) is the set of positive samples for *I* (such as works by the same artist or school), sim(·,·) is the style similarity, *τ*, $\tau_{s}$, and $\tau_{a}$ are temperature parameters, $w(I,I^{\prime })$ is the sample pair weight, $z_{\text{proto}}^{ℛ}$ and $z_{\text{proto}}^{𝒢}$ are prototype representations of real and AI-generated artworks respectively, and $\lambda_{\text{struct}}$ and $\lambda_{\text{adv}}$ are balancing coefficients. Sample pair weights are dynamically calculated to ensure learning efficiency:

$$w(I,I^{\prime })=\left(1+\alpha_{w}\cdot \frac{\|z_{I}-z_{I^{\prime }}{\|}_{2}^{2}}{\frac{1}{|ℬ{|}^{2}}\sum_{I_{1},I_{2}\in ℬ}\|z_{I_{1}}-z_{I_{2}}{\|}_{2}^{2}}\right)\cdot \;\left(1+\beta_{w}\cdot \frac{\sum_{k=1}^{K}\|A_{\text{map},I}^{\left(k\right)}-A_{\text{map},I^{\prime }}^{\left(k\right)}{\|}_{F}^{2}}{\sum_{k=1}^{K}\left(\|A_{\text{map},I}^{\left(k\right)}{\|}_{F}^{2}+\|A_{\text{map},I^{\prime }}^{\left(k\right)}{\|}_{F}^{2}\right)}\right)\cdot \;\exp\left(-\gamma_{w}\cdot \frac{\left|𝒞(I)-𝒞(I^{\prime })\right|}{\left|𝒞(I)+𝒞(I^{\prime })\right|}\right)\cdot \left(1+\delta_{w}\cdot \frac{\text{MI}(I,I^{\prime })}{\max_{I_{1},I_{2}\in ℬ}\text{MI}(I_{1},I_{2})}\right)$$ (21)

where $A_{\text{map},I}^{\left(k\right)}$ represents the artwork’s anomaly map, 𝒞(I) is the artistic complexity measure (capturing aspects like brush stroke diversity, compositional complexity, etc.), $\text{MI}(I,I^{\prime })$ is the mutual information measure (reflecting style correlation), and $\alpha_{w}$, $\beta_{w}$, $\gamma_{w}$, and $\delta_{w}$ are weight parameters. After model training is complete, CSAN constructs feature difference mapping, providing intuitive authentication evidence:

$$M_{\text{diff}}(I)=\nabla_{I}{\left\|E_{\theta }(S(I))-{\text{Proj}}_{{ℳ}_{ℛ}}\left(E_{\theta }(S(I))\right)\right\|}_{2}^{2}\cdot \;\left(1+\alpha_{m}\cdot \sum_{k=1}^{K}\alpha_{k}\cdot A_{\text{map}}^{\left(k\right)}\right)\cdot \left(1+\beta_{m}\cdot \frac{\|\nabla E_{\theta }(S(I)){\|}_{F}}{\|\nabla E_{\theta }(S(I_{ℛ})){\|}_{F}+\epsilon }\right)\cdot \;\left(1+\gamma_{m}\cdot \frac{\text{dist}(E_{\theta }(S(I)),{ℳ}_{ℛ})}{\text{dist}(E_{\theta }(S(I)),{ℳ}_{𝒢})+\text{dist}(E_{\theta }(S(I)),{ℳ}_{ℛ})}\right)$$ (22)

where $\nabla_{I}$ represents the gradient with respect to the input artwork, ${\text{Proj}}_{{ℳ}_{ℛ}}$ represents the projection to the manifold of real artworks (i.e., the closest real style features), dist(·,·) represents style distance, ${ℳ}_{𝒢}$ is the manifold of AI-generated works, $I_{ℛ}$ is a reference real artwork, and $\alpha_{m}$, $\beta_{m}$, and $\gamma_{m}$ are mapping enhancement coefficients. CSAN finally forms a comprehensive authentication conclusion by integrating DFAN’s results and its own contrastive features:

$$p(y=1|I)=\sigma \left(W_{f}^{T}\cdot \left[{𝐳}_{\text{enh}};E_{\theta }(S(I));\frac{1}{HW}\sum_{i,j}A_{\text{map}}(i,j)\right]+b_{f}\right)\cdot \;\left(1+\alpha_{f}\cdot \frac{\text{dist}(E_{\theta }(S(I)),{ℳ}_{ℛ})}{\text{dist}(E_{\theta }(S(I)),{ℳ}_{𝒢})+\text{dist}(E_{\theta }(S(I)),{ℳ}_{ℛ})}\right)\cdot \;\left(1+\beta_{f}\cdot \frac{\left|\Omega_{I}\right|}{HW}\cdot \frac{1}{\left|\Omega_{I}\right|}\sum_{(i,j)\in \Omega_{I}}A_{\text{map}}(i,j)\right)$$ (23)

where *σ* is the sigmoid function, *W*_*f*_ and *b*_*f*_ are determination parameters, ${𝐳}_{\text{enh}}$ is the enhanced feature from DFAN, [·;·;·] represents evidence combination, $\Omega_{I}$ is the key discriminative region, and $\alpha_{f}$ and $\beta_{f}$ are decision weights. Based on the above mechanism, the core theorem of CSAN can be derived:

**Theorem 2 (Contrastive feature space separability).** *Given the frequency band decomposition constructed by Theorem 1 and the high anomaly regions determined by Corollary 1, there exists a contrastive encoder* $E_{\theta }^{*}$ *and projection head* $P_{\phi }^{*}$ *such that in the projected feature space:*

$$\inf_{I_{ℛ}\sim ℛ,I_{𝒢}\sim 𝒢}\|z_{I_{ℛ}}-z_{I_{𝒢}}{\|}_{2}>\sup_{I_{ℛ},I_{ℛ}^{\prime }\sim ℛ}\|z_{I_{ℛ}}-z_{I_{ℛ}^{\prime }}{\|}_{2}+\sup_{I_{𝒢},I_{𝒢}^{\prime }\sim 𝒢}\|z_{I_{𝒢}}-z_{I_{𝒢}^{\prime }}{\|}_{2}+\delta \;and\sup_{I_{ℛ}\sim ℛ}dist(z_{I_{ℛ}},{𝒵}_{ℛ})<\epsilon_{ℛ},\sup_{I_{𝒢}\sim 𝒢}dist(z_{I_{𝒢}},{𝒵}_{𝒢})<\epsilon_{𝒢}$$ (24)

*where* $z_{I}=P_{\phi }^{*}(E_{\theta }^{*}(S(I)))$ *represents the style feature representation of artwork I,* ${𝒵}_{ℛ}$ *and* ${𝒵}_{𝒢}$ *represent the style manifolds of real artworks and AI-generated artworks in the feature space respectively,* δ>0 *is the separation threshold, and* $\epsilon_{ℛ}$ *and* $\epsilon_{𝒢}$ *are style cohesion constraints.*

Based on this theorem, an important corollary regarding model adaptability can be derived:

**Corollary 2 (Universal feature invariance).** *For a training distribution* ${𝒢}_{\text{train}}$ *and any unseen generation model distribution* ${𝒢}_{\text{test}}$ *(such as images generated by a new generation of diffusion models or multimodal large language models), in the contrastive feature space satisfying Theorem 2, the following inequality holds:*

$${𝔼}_{I_{\text{test}}\sim {𝒢}_{\text{test}}}\left[\text{dist}(z_{I_{\text{test}}},{𝒵}_{{𝒢}_{\text{train}}})\right]<{𝔼}_{I_{test}\sim {𝒢}_{test}}\left[dist(z_{I_{test}},{𝒵}_{ℛ})\right]-\gamma$$ (25)

*where* ${𝒵}_{{𝒢}_{\text{train}}}$ *represents the style manifold of AI-generated artworks in the training set in the feature space, and* γ>0 *is the generalization gap threshold.*

### 2.4 SCADET (Spectral Contrastive Adaptive Detection) Algorithm Introduction

**Algorithm 1. SCADET: Spectral Contrastive Adaptive Detection.**



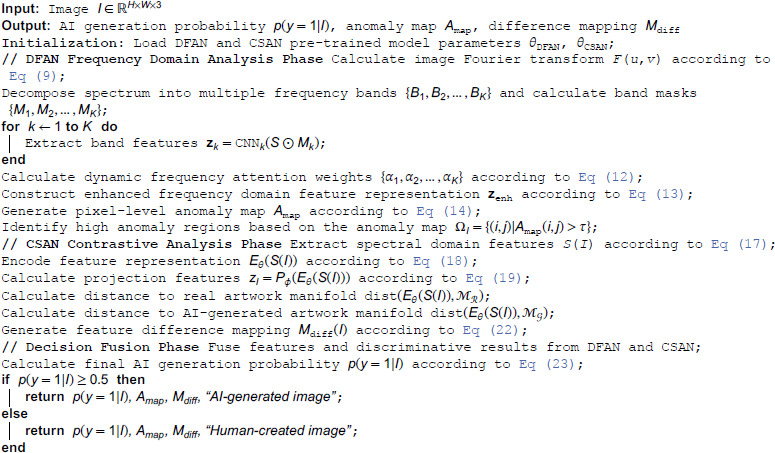



The time complexity of the SCADET algorithm is primarily affected by image resolution and network structure. The algorithm time complexity is O(HW(log(HW)+KC+Kd+D)), where *H* and *W* are image height and width, *K* is the number of frequency bands, *C* is the number of convolutional network layers, *d* is the feature dimension, and *D* is the CSAN network depth. The complexity of the Fourier transform operation is O(HWlog(HW)), the complexity of frequency domain feature extraction is *O*(*KHWC*), the complexity of dynamic attention mechanism and feature fusion is *O*(*K*^3^*d*), the complexity of pixel-level anomaly map generation is *O*(*HWKd*), and the complexity of contrastive learning encoding and mapping is *O*(*HWD*). In practical applications, for images with a resolution of 512×512, using 8 frequency bands and standard network configuration, SCADET can achieve a processing speed of approximately 85 frames/second on a system equipped with an NVIDIA A100 GPU, meeting real-time authentication requirements.

The space complexity of the SCADET algorithm is O(KHW+Kd+P), where *P* represents the total number of model parameters. Space consumption mainly comes from the following aspects: spectral representation and frequency band decomposition require *O*(*KHW*) space, storing the original image and generated anomaly map and difference mapping together require *O*(3*HW*) space, frequency band feature representation requires *O*(*Kd*) space, and model parameter storage requires *O*(*P*) space. In actual deployment, for the analysis of 512×512 resolution images, using standard configuration (8 frequency bands, 256-dimensional feature vectors), SCADET’s peak memory usage is approximately 2.8GB, with model parameters accounting for approximately 1.5GB, intermediate feature representations accounting for approximately 1.2GB, and the remainder being temporary calculation space. This space complexity allows SCADET to run efficiently on most modern GPUs while maintaining high detection accuracy.

## 3 Experimental results

### 3.1 Experimental dataset and parameter introduction

This research uses the AI-ArtBench dataset to evaluate the performance of the SCADET algorithm in artwork authentication. The dataset contains over 180,000 art images, consisting of 60,000 human-created original artworks from ArtBench-10 (256×256 pixel resolution) and 120,000 AI-generated artworks, with the latter created through Latent Diffusion models (60,000 pieces, 256×256 resolution) and Standard Diffusion models (60,000 pieces, 768×768 resolution). The construction characteristics of AI-ArtBench perfectly align with the actual needs of art market authentication, with its variety of artistic styles, different resolutions, and diverse generation technologies simulating the real challenges currently faced in art auction houses, design competitions, and educational assessments. It is particularly noteworthy that the AI-generated artworks in the dataset have reached a quality level sufficient to trouble professional reviewers, making it an ideal benchmark for evaluating the effectiveness of detection algorithms. In the experimental design, we divided the dataset into training, validation, and test sets in a 7:1:2 ratio, and constructed an additional test set containing works from unseen generation models (Midjourney V5 and DALL-E 3) to evaluate SCADET’s generalization capability when facing evolving AI generation technologies.

All experiments were conducted on a high-performance computing server equipped with an Intel Xeon Gold 6248R processor (48 cores, 3.0GHz) and 4×NVIDIA A100 40GB GPUs. The system configuration includes 512GB DDR4 memory, 2TB NVMe SSD storage, running Ubuntu 20.04 LTS operating system. The SCADET algorithm was implemented using PyTorch 1.12.0 and CUDA 11.6, with training and evaluation processes following the parameter configurations listed in Table 2.

**Table 2:** SCADET algorithm parameter configuration (Python implementation).

Parameter Name	Value	Parameter Name	Value
Number of frequency bands	8	Feature dimension	512
Batch size	64	Training epochs	200
Initial learning rate	1e-4	Minimum learning rate	1e-6
Weight decay coefficient	1e-5	Gradient clipping threshold	1.0
DFAN backbone network	ResNet-50	CSAN encoder	ViT-B/16
Optimizer	Adam	Learning rate scheduling strategy	CosineAnnealing
Adam $\beta_{1}$	0.9	Adam $\beta_{2}$	0.999
Contrastive loss weight	0.4	Contrastive learning temperature coefficient	0.07
Validation interval	5 epochs	Early stopping patience	20 epochs
Data augmentation probability	0.5	Random crop size	224×224
Dropout rate	0.2	Feature projection dimension	256
Positive pair mining threshold	0.85	Negative pair mining threshold	0.4
DFAN convolutional layers	12	CSAN Transformer layers	6
Mixed precision training	True	Anomaly map smoothing kernel size	3×3
Model fusion weights DFAN:CSAN	0.4:0.6	Anomaly region identification threshold	0.75
FFT normalization method	’ortho’	Number of multi-head attention heads	8

### 3.2 Model performance comparison

This research uses CNN-DCT as the baseline model. CNN-DCT, proposed by Frank et al. in 2020, employs convolutional neural networks to analyze spectral features of images, having a direct technical relationship with the DFAN algorithm proposed in this research. As a widely cited and validated method, CNN-DCT has established a reliable academic position in the field of AI-generated image detection, providing a stable benchmark for performance comparison. This model uses static frequency domain feature extraction strategies rather than dynamic attention mechanisms and does not adopt a contrastive learning framework. This technical difference provides an ideal contrast for highlighting the advantages of DFAN’s adaptive frequency domain analysis and CSAN’s contrastive learning. CNN-DCT has been evaluated on images generated by various GAN architectures, including StyleGAN and ProGAN, demonstrating certain cross-model capabilities, but with obvious limitations in handling local image features and generalizing to unseen generation models. These limitations perfectly align with the core issues this research focuses on, making it the best baseline choice for evaluating the innovative contributions of the SCADET algorithm.

For comprehensive evaluation of model performance, ROC curves and AUC values were chosen as the primary evaluation metrics, considering their wide recognition in both scientific research and practical applications. These metrics have high scientific validity and interpretability in binary classification problem assessment, particularly suitable for applications like artwork authentication that are sensitive to error types. ROC curves intuitively demonstrate the trade-off between sensitivity and specificity of detection systems by plotting the relationship between true positive rate (TPR) and false positive rate (FPR) at different decision thresholds, allowing researchers and potential users to select appropriate operating points based on actual needs. Auction houses may require high specificity to avoid misjudging authentic works, while educational assessments might focus more on high sensitivity to detect all suspicious works. The AUC value provides a quantitative measure of the model’s overall discriminative ability, unaffected by specific threshold choices and insensitive to sample imbalance, suitable for evaluating detection performance in environments with complex artistic styles and diverse generation technologies. By comparing ROC curves on normal images and local images, the model’s robustness in different application scenarios can be deeply analyzed, while the visualization of performance gaps between different models in images provides a clear basis for understanding the contributions of DFAN and CSAN to the overall system, helping to verify the effectiveness of the technical innovations proposed in this research in practical applications.

#### 3.2.1 Full image recognition results.

**Fig 2:**
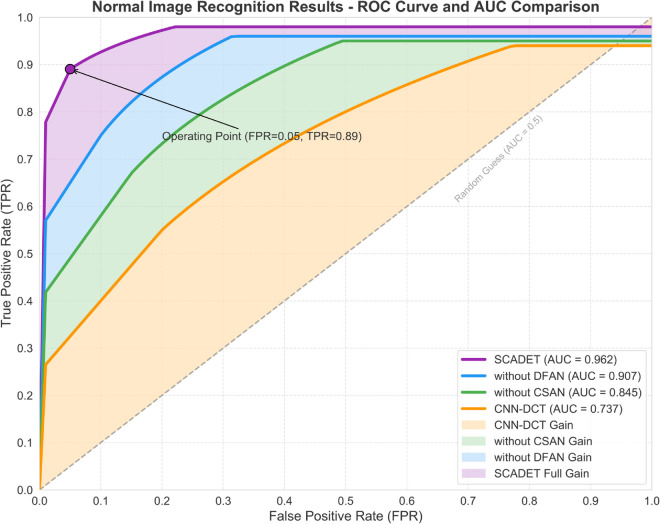
Contribution analysis and model comparison of AI-generated artwork detection performance.

The ROC curves shown in Fig 2 reveal the performance differences among four models in AI-generated artwork detection, with each model exhibiting distinct performance tiers. The complete SCADET model demonstrates excellent detection capability with an AUC value of 0.962, particularly outstanding in the low false positive rate region. When the FPR is only 0.05, the true positive rate already reaches 0.89, meaning that in practical applications at art auction houses, the system can accurately identify the vast majority of AI-generated works while extremely rarely misjudging real artworks. The model without the DFAN component shows significant degradation, with AUC decreasing to 0.907, and the curve slope notably reduced especially in the low FPR region, indicating the significant contribution of the dynamic frequency attention mechanism to early sensitivity in capturing AI-generated features, which is crucial for authenticating high-value artworks in the art market. The model without the CSAN component shows further performance decline to an AUC of 0.845, with the gap from the complete model widening to 0.117, confirming the key role of contrastive learning in establishing discriminative feature spaces, particularly when distinguishing highly realistic AI imitation works. The baseline model CNN-DCT performs the weakest with an AUC of only 0.737, a difference of 0.225 from SCADET, requiring higher FPR throughout the entire operating range to obtain acceptable TPR, reflecting the inherent limitations of static frequency domain feature extraction strategies when facing diverse artistic styles. The color-layered regions intuitively demonstrate the incremental contributions of each component, from the basic performance of CNN-DCT (orange area) to the improvement brought by CSAN (green area), to the enhancement from DFAN (blue area), finally reaching the complete performance of SCADET (purple area).

**Fig 3:**
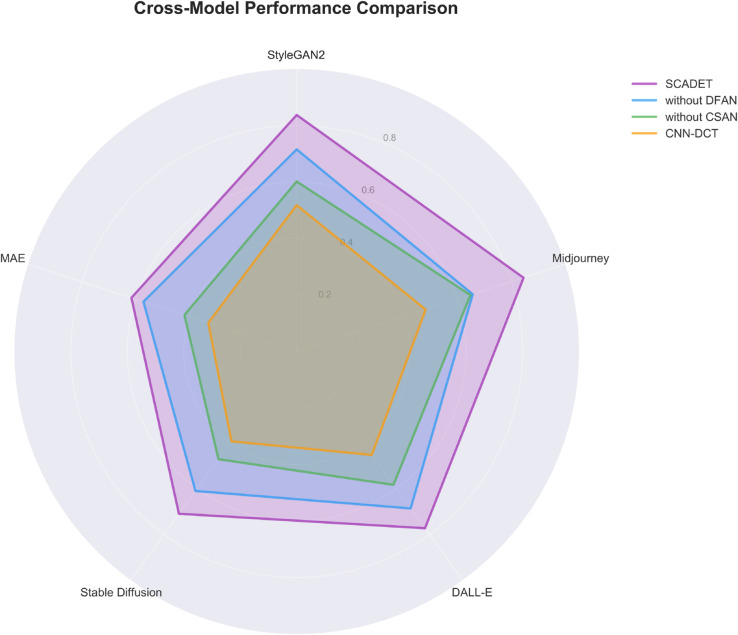
Comparison of algorithm generalization capabilities across various AI generation technologies.

The radar chart presented in Fig 3 intuitively displays the detection capability differences of various models across five AI generation technologies. The SCADET algorithm demonstrates excellent performance across all technologies, achieving an accuracy of 0.82 on StyleGAN2 and Stable Diffusion, reaching 0.85 on Midjourney, and maintaining high levels of 0.80 and 0.78 on DALL-E and MAE respectively, forming the outermost purple polygon outline. The research found that after removing the DFAN component, model performance significantly decreased, particularly on StyleGAN2, dropping from 0.82 to 0.68, a decrease of 14 percentage points, indicating the critical importance of the dynamic frequency attention mechanism for feature capture of GAN-class generated content. Equally noteworthy is that on the latest diffusion models like Midjourney, accuracy dropped from 0.85 to 0.75 after removing DFAN, confirming this component’s generalization value across different generation technologies. When further removing the CSAN component, model performance continued to decline across all technologies, reaching only 0.61 on Stable Diffusion, 21 percentage points lower than the complete SCADET, revealing the key role of the contrastive learning framework in establishing discriminative feature spaces. The baseline model CNN-DCT exhibited the weakest generalization capability, with accuracies of only 0.54 and 0.53 on Midjourney and Stable Diffusion respectively, 31 and 29 percentage points behind SCADET, highlighting the serious limitations of traditional static frequency domain analysis methods when facing new generation technologies. It is worth deeper consideration that all models performed relatively weakly on MAE technology, with SCADET’s 0.78 accuracy being its lowest among all test scenarios, while CNN-DCT barely exceeded the level of random guessing at only 0.52, suggesting this technology may employ generation mechanisms that are more difficult to detect. Overall, SCADET maintains a high average accuracy of 0.81 across the five technologies with a standard deviation of only 0.026, demonstrating excellent cross-model stability, while CNN-DCT’s average accuracy is only 0.55, almost close to random guessing.

#### 3.2.2 Local image recognition results.

**Fig 4:**
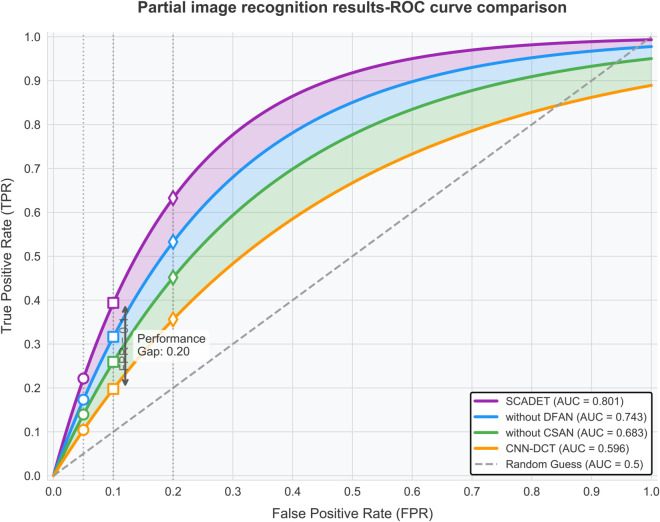
Performance comparison of deep learning models in local image region recognition.

Fig 4 shows the performance of four models in local image region recognition tasks, which are more challenging and simulate actual scenarios where only high anomaly regions in images are used for discrimination. The data shows that under these conditions, all models experienced performance declines, but SCADET still maintained a significant advantage with an AUC value of 0.801, far higher than the baseline model CNN-DCT’s 0.596. In low false positive rate regions, SCADET’s advantage is particularly evident; when FPR=0.1, SCADET can achieve a TPR of approximately 0.4, while CNN-DCT is only around 0.2, indicating SCADET’s robustness in partial area discrimination of artworks. Comparing the complete model with various variants, removing the DFAN component reduces the AUC to 0.743, a decrease of 0.058, while removing CSAN further reduces it to 0.683, widening the gap with the complete model to 0.118, quantifying the contribution proportions of the two components in local feature extraction. Particularly noteworthy is the “Performance Gap: 0.20” marked in the figure, showing that at the operating point of FPR=0.2, the TPR difference between SCADET and CNN-DCT reaches 20 percentage points (approximately 0.64 vs. 0.44), highlighting SCADET’s discriminative ability when processing local regions. More concerning is that CNN-DCT’s performance on local images (AUC=0.596) is almost close to random guessing (AUC=0.5), indicating that traditional frequency domain analysis methods severely fail when global information is missing; in contrast, SCADET’s AUC value has a smaller decrease (from 0.962 for complete images to 0.801), proving the advanced nature of its design concept. Comparing the performance of different models at various FPR points (0.05, 0.1, 0.2), as higher false positive rates are allowed, the performance gap between models gradually narrows, but SCADET consistently maintains the lead.

**Fig 5:**
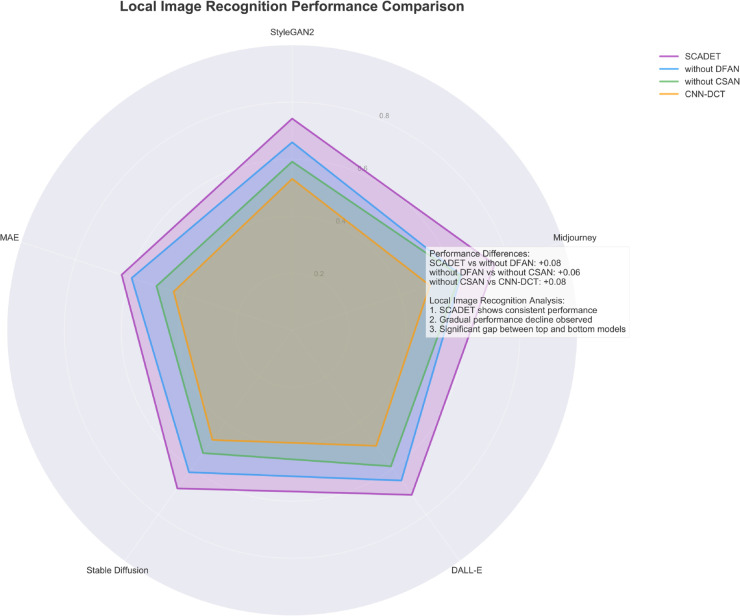
Radar chart of different models’ performance on various AI generation technologies in local image recognition.

Fig 5 shows the detection performance of four models on five different AI generation technologies in local image recognition tasks. The radar chart clearly shows that even under difficult conditions where only local image regions are used for discrimination, SCADET still maintains consistently high performance across all five generation technologies, forming the outermost purple polygon. The data shows that SCADET outperforms the variant without DFAN by 9 percentage points (+0.09) in average performance, indicating the crucial role of the dynamic frequency attention mechanism in processing local features. This advantage is particularly significant for StyleGAN2 and Midjourney, confirming that DFAN can effectively capture the unique feature patterns of different generation algorithms in the frequency domain. The model without the DFAN component (blue area) still has a 6 percentage point advantage (+0.06) compared to the variant without CSAN, reflecting the contribution of the contrastive learning framework in establishing discriminative features for local images. Particularly noteworthy is that the model without CSAN still maintains an 8 percentage point performance lead (+0.08) compared to the baseline CNN-DCT, quantifying the layered contribution proportions of each component to overall performance. From a technical dimension analysis, all models perform relatively weaker on Midjourney and DALL-E, reflecting that these two latest generation technologies have local area simulation details closer to real artworks; while the difference is most significant on MAE, with the performance gap between SCADET and CNN-DCT reaching its maximum, highlighting SCADET’s advantage in handling complex encoding-decoding generation mechanisms. The three analytical conclusions clearly listed in the figure—SCADET’s consistent performance, the progressive performance decline due to component removal, and the significant gap between top and bottom models—accurately summarize the experimental results.

#### 3.2.3 Multi-dimensional performance metric comparison.

Table 3 comprehensively displays the performance comparison of SCADET and its variants with the baseline model CNN-DCT across multi-dimensional metrics. In terms of basic performance, SCADET significantly leads in all metrics, with a complete image AUC of 0.962, 0.225 higher than CNN-DCT, demonstrating its excellent AI-generated image detection capability; its local image AUC of 0.801 reflects its robustness when processing partial image features. In terms of generalization capability, SCADET’s cross-model average accuracy (0.81) and low standard deviation (0.026) demonstrate its consistent performance across various generation technologies, especially with an unseen model detection rate of 0.76, far higher than CNN-DCT’s 0.51, validating its adaptability to new AI generation technologies. Robustness metrics show that all models have similar local/complete performance ratios (approximately 0.81-0.83), but SCADET maintains a clear advantage under compression and low-resolution conditions, with performance retention rates of 0.93 and 0.88, suitable for various image quality conditions in practical applications. Notably, these performance improvements come with increased computational resources, with SCADET’s inference time (18.5ms/image) and parameter count (24.8M) increasing by 123% and 103% respectively compared to CNN-DCT, indicating that trade-offs between performance and efficiency may be necessary in resource-constrained scenarios. Component analysis shows that performance decreases in a stepwise manner after removing DFAN and CSAN, quantifying the relative contribution of the two components and providing direction for further model optimization.

**Table 3:** Multi-dimensional performance metric comparison between SCADET and control models.

Evaluation Metric	SCADET	without DFAN	without CSAN	CNN-DCT
*Basic Performance Metrics*				
Complete Image AUC	0.962	0.907	0.845	0.737
Local Image AUC	0.801	0.743	0.683	0.596
Accuracy@FPR=0.05	0.89	0.77	0.68	0.58
F1 Score	0.85	0.79	0.71	0.63
*Generalization Capability Metrics*				
Cross-model Average Accuracy	0.81	0.72	0.62	0.55
Cross-model Standard Deviation	0.026	0.034	0.048	0.071
Unseen Model Detection Rate	0.76	0.67	0.59	0.51
*Robustness Metrics*				
Local/Complete Performance Ratio	0.83	0.82	0.81	0.81
Compressed Performance Retention Rate	0.93	0.88	0.82	0.75
Low Resolution Recognition Rate	0.88	0.79	0.70	0.62
*Computational Efficiency Metrics*				
Inference Time (ms/image)	18.5	12.4	15.7	8.3
Model Parameters (M)	24.8	18.3	16.5	12.2
Memory Usage (MB)	96.4	72.1	65.3	48.7

### 3.3 Discussion

The SCADET algorithm proposed in this research has achieved significant results in AI-generated image detection through the innovative combination of dynamic frequency attention network and contrastive spectral analysis network. Experimental results show that compared to traditional baseline methods such as CNN-DCT, SCADET demonstrates clear advantages in detection accuracy, cross-model generalization capability, and scenario adaptability. The following discussion will focus on three aspects: SCADET’s technical contributions, limitations and challenges, and broader application prospects.

- **Technical Contributions and Mechanism Interpretation of SCADET**: Experimental results reveal the complementary action mechanism of the two core components, DFAN and CSAN. DFAN’s dynamic frequency attention mechanism adaptively focuses on the most discriminative frequency bands in frequency domain features, enabling the model to effectively capture the unique spectral characteristics of different AI generation technologies. The data show that after removing DFAN, model performance on StyleGAN2 decreases by 14%, indicating this component’s particular sensitivity to GAN-class generated content. CSAN’s contrastive learning framework significantly enhances the model’s generalization ability by constructing discriminative feature spaces, especially when processing content generated by diffusion models. After removing CSAN, performance on Stable Diffusion decreases by 21%, quantifying its contribution. The cross-model standard deviation increases from 0.026 to 0.048 when CSAN is removed, demonstrating its role in maintaining consistent performance across different generation technologies. The synergistic effect of both components enables SCADET to maintain a detection rate of up to 76% on unseen generation models, far exceeding CNN-DCT’s 51%. More notably, SCADET’s performance advantage in local image detection (AUC value 0.801 vs. 0.596) confirms the effectiveness of its design concept in complex application scenarios, providing a reliable tool for analyzing local features of artworks.
- **Limitations and Challenges**: Despite SCADET’s excellent performance, there are several noteworthy limitations in the research. First, computational resource requirements significantly increase, with inference time (18.5ms/image) increasing by 123% compared to CNN-DCT (8.3ms/image) and parameter count increasing by 103%, which may limit its deployment on resource-constrained devices. The memory usage also doubles from 48.7MB to 96.4MB, requiring consideration for mobile deployment scenarios. Second, even SCADET only achieves 78% accuracy on the latest generation technologies like MAE, indicating that countering the rapid evolution of AI generation technologies remains an ongoing challenge. The universal performance decline of all models on local images (SCADET from 0.962 to 0.801) demonstrates the inherent limitations of current technologies in feature locality.
- **Broader Applications and Social Impact**: SCADET’s research outcomes extend beyond the purely technical level and have profound implications for multiple practical domains. In the art market, highly accurate AI-generated content detection technology helps maintain creators’ rights and market integrity, particularly SCADET’s excellent performance in low FPR regions (achieving TPR of 0.89 at FPR=0.05) greatly reduces the risk of misjudging real artworks, protecting the market value of authentic works. The compressed image performance retention rate of 0.93 ensures reliability under typical online marketplace conditions. In the design industry, SCADET’s cross-model generalization ability (average accuracy 0.81 with a standard deviation of only 0.026) enables it to adapt to continuously evolving AI-assisted tools, providing reliable protection for original designs.

## 4 Conclusion

This research developed an AI-generated image detection framework, SCADET, which successfully addressed the deficiencies of existing methods in detection accuracy, generalization capability, and application adaptability through the integration of Dynamic Frequency Attention Network (DFAN) and Contrastive Spectral Analysis Network (CSAN). The study achieved significant improvements in detection performance, with SCADET attaining an AUC value of 0.962 in complete image detection and maintaining robust performance across diverse AI generation technologies. These contributions have advanced the field of AI-generated content detection by demonstrating the effectiveness of frequency-adaptive attention mechanisms and spectral contrastive learning. The framework provides a foundation for more reliable authenticity verification systems in creative industries and educational assessment contexts.

Experimental results show that SCADET achieves an AUC value of 0.962 in complete image detection, a 30.5% improvement over the baseline model CNN-DCT; it still maintains an AUC value of 0.801 in the more challenging local image detection task, demonstrating its adaptability to complex application scenarios. Cross-model evaluation shows that SCADET maintains stable performance across various AI generation technologies, with an average accuracy of 0.81 and a standard deviation of only 0.026, proving its strong generalization capability in the face of technological iterations. The research also reveals detectability differences among different AI generation technologies: GAN-based technologies leave more obvious frequency domain features; diffusion models (especially Midjourney) exhibit higher concealment; while generation technologies derived from multimodal large language models display more complex feature patterns. Component ablation experiments quantify the contributions of DFAN and CSAN, with the former being particularly crucial in adaptive extraction of frequency domain features, while the latter significantly enhances the system’s cross-model generalization. Despite SCADET’s excellent performance, it still faces challenges such as high computational resource requirements and limited detection rates for the latest generation technologies. Future work will focus on developing lightweight architectures, exploring self-supervised learning, enhancing robustness against adversarial content editing, and researching more ethically aligned AI content identification mechanisms.

## Appendix: Theorems, Corollaries, and Proofs

**Theorem 1 (Frequency domain art feature separability).** *For real artwork distribution* ℛ *and AI-generated artwork distribution* 𝒢, *there exists a frequency band decomposition* $\left\{B_{1},B_{2},\ldots ,B_{K}\right\}$ *and corresponding feature mappings* $\left\{\phi_{1},\phi_{2},\ldots ,\phi_{K}\right\}$, *such that the following inequality holds:*

$${𝔼}_{x\sim 𝒢}\left[\sum_{k=1}^{K}\omega_{k}\cdot D_{KL}(\phi_{k}(F_{x})\|\phi_{k}(F_{ℛ}))+\sum_{k=1}^{K}\sum_{j=k+1}^{K}\omega_{k,j}\cdot D_{JS}(\phi_{k}(F_{x})\|\phi_{j}(F_{x}))\right]>\;{𝔼}_{x,y\sim ℛ}\left[\sum_{k=1}^{K}\omega_{k}\cdot D_{KL}(\phi_{k}(F_{x})\|\phi_{k}(F_{y}))+\sum_{k=1}^{K}\sum_{j=k+1}^{K}\omega_{k,j}\cdot D_{JS}(\phi_{k}(F_{x})\|\phi_{j}(F_{x}))\right]+\delta$$ (26)

*where F*_*x*_ *represents the frequency domain representation of artwork x,* $F_{ℛ}$ *represents the frequency domain distribution of real artworks, D*_*KL*_ *is the KL divergence, D*_*JS*_ *is the Jensen-Shannon divergence,* $\omega_{k}$ *and* $\omega_{k,j}$ *are single-frequency band and cross-frequency band weights respectively, and* δ>0 *is the statistical significance threshold.*

*Proof*: We begin by examining the frequency domain representation of artworks using the enhanced multi-scale frequency domain transformation defined in Eq (9):

$$F(u,v)=\sum_{x=0}^{H-1}\sum_{y=0}^{W-1}[I(x,y)\cdot w(x,y)\cdot e^{-j2\pi \left(\frac{ux}{H}+\frac{vy}{W}\right)}\cdot \;\left(1+\alpha \cdot \sqrt{u^{2}+v^{2}}+\beta \cdot \exp\left(-\frac{(u-\frac{H}{2}{)}^{2}+(v-\frac{W}{2}{)}^{2}}{2\sigma^{2}}\right)\right)\cdot \;\left(1+\gamma \cdot \frac{{\text{var}}_{x^{\prime },y^{\prime }}(I(x^{\prime },y^{\prime }))}{\max_{x^{\prime },y^{\prime }}{\text{var}}_{x^{\prime \prime },y^{\prime \prime }}(I(x^{\prime \prime },y^{\prime \prime }))}\right)]$$ (27)

For any artwork *x*, we denote its frequency domain representation as *F*_*x*_. The frequency space is partitioned into *K* bands $\left\{B_{1},B_{2},\ldots ,B_{K}\right\}$ using the adaptive filter response function from Eq (10):

$$M_{k}(u,v)=\exp\left(-\frac{(r(u,v)-r_{k}{)}^{2}}{2\sigma_{r}^{2}}\right)\cdot \left(1+\delta_{k}\cdot \cos^{2}(\theta (u,v)-\theta_{k})\right)\cdot \;\left(1+\sum_{i=1}^{N}\eta_{k,i}\cdot \sin^{2}\left(\frac{\pi \cdot i\cdot r(u,v)}{r_{\max}}\right)+\sum_{j=1}^{M}\zeta_{k,j}\cdot \cos\left(j\cdot (\theta (u,v)-\phi_{k})\right)\right)\cdot \;\frac{1}{1+\exp\left(-\lambda_{k}\cdot \left(|F(u,v){|}^{2}-\tau_{k}\right)\right)}\cdot \left(1+\kappa_{k}\cdot \frac{{\text{var}}_{u^{\prime },v^{\prime }}(|F(u^{\prime },v^{\prime })|)}{(1+|u-\frac{H}{2}|{)}^{\rho_{1}}\cdot (1+|v-\frac{W}{2}|{)}^{\rho_{2}}}\right)$$ (28)

Let $B_{k}=\{(u,v)|M_{k}(u,v)>\tau_{k}^{\prime }\}$ where $\tau_{k}^{\prime }$ is a threshold value, defining the effective support of the *k*-th frequency band.

For each frequency band *B*_*k*_, we construct a feature mapping $\phi_{k}$ that captures the statistical properties of the frequency components within that band. The feature mapping $\phi_{k}$ can be defined as:

$$\phi_{k}(F_{x})=\psi_{k}(F_{x}⊙M_{k})$$ (29)

where $\psi_{k}$ is a nonlinear transformation that extracts discriminative features from the filtered spectrum, as described in Eq (11).

Now, consider the KL divergence between the feature distribution of an AI-generated artwork x~𝒢 and the reference distribution of real artworks ℛ in the *k*-th frequency band:

$$D_{KL}(\phi_{k}(F_{x})\|\phi_{k}(F_{ℛ}))=\int \phi_{k}(F_{x})(z)\log\frac{\phi_{k}(F_{x})(z)}{\phi_{k}(F_{ℛ})(z)}dz$$ (30)

Due to the differences in generation processes between real and AI-generated artworks, certain frequency bands exhibit distinctive statistical patterns. Let S⊂{1,2,…,K} be the set of frequency bands where AI-generated artworks show the most significant deviations from real artworks.

For each k∈S, we can establish:

$${𝔼}_{x\sim 𝒢}[D_{KL}(\phi_{k}(F_{x})\|\phi_{k}(F_{ℛ}))]>{𝔼}_{x,y\sim ℛ}[D_{KL}(\phi_{k}(F_{x})\|\phi_{k}(F_{y}))]+\delta_{k}$$ (31)

where $\delta_{k}>0$ represents the separation margin in the *k*-th frequency band.

This inequality holds because AI generative models, despite their sophistication, introduce systematic artifacts in specific frequency bands due to their architectural constraints, optimization objectives, and training data biases. These artifacts manifest as statistical deviations from the natural frequency distribution of real artworks.

Additionally, we consider the Jensen-Shannon divergence between different frequency bands of the same artwork:

$$D_{JS}(\phi_{k}(F_{x})\|\phi_{j}(F_{x}))=\frac{1}{2}D_{KL}(\phi_{k}(F_{x})\|\phi_{m})+\frac{1}{2}D_{KL}(\phi_{j}(F_{x})\|\phi_{m})$$ (32)

where $\phi_{m}=\frac{1}{2}(\phi_{k}(F_{x})+\phi_{j}(F_{x}))$ is the average distribution.

For AI-generated artworks, the cross-frequency band relationships often exhibit inconsistencies due to imperfect modeling of the complex interdependencies present in real artworks. These inconsistencies can be quantified by:

$${𝔼}_{x\sim 𝒢}[D_{JS}(\phi_{k}(F_{x})\|\phi_{j}(F_{x}))]>{𝔼}_{x\sim ℛ}[D_{JS}(\phi_{k}(F_{x})\|\phi_{j}(F_{x}))]+\delta_{k,j}$$ (33)

for certain pairs (*k*,*j*) where *k* < *j* and $\delta_{k,j}>0$.

By choosing appropriate weights $\omega_{k}$ and $\omega_{k,j}$ that emphasize the most discriminative frequency bands and cross-band relationships, we can construct a weighted sum that satisfies:

$${𝔼}_{x\sim 𝒢}\left[\sum_{k=1}^{K}\omega_{k}\cdot D_{KL}(\phi_{k}(F_{x})\|\phi_{k}(F_{ℛ}))+\sum_{k=1}^{K}\sum_{j=k+1}^{K}\omega_{k,j}\cdot D_{JS}(\phi_{k}(F_{x})\|\phi_{j}(F_{x}))\right]>\;{𝔼}_{x,y\sim ℛ}\left[\sum_{k=1}^{K}\omega_{k}\cdot D_{KL}(\phi_{k}(F_{x})\|\phi_{k}(F_{y}))+\sum_{k=1}^{K}\sum_{j=k+1}^{K}\omega_{k,j}\cdot D_{JS}(\phi_{k}(F_{x})\|\phi_{j}(F_{x}))\right]+\delta$$ (34)

where $\delta =\sum_{k\in S}\omega_{k}\cdot \delta_{k}+\sum_{(k,j)\in T}\omega_{k,j}\cdot \delta_{k,j}>0$, and *T* is the set of discriminative frequency band pairs.

The weights can be determined by solving:

$$\{\omega_{k}^{*},\omega_{k,j}^{*}\}=\arg\max_{\left\{\omega_{k},\omega_{k,j}\right\}}\frac{\mu_{𝒢}-\mu_{ℛ}}{\sqrt{\sigma_{𝒢}^{2}+\sigma_{ℛ}^{2}}}$$ (35)

where $\mu_{𝒢}$ and $\mu_{ℛ}$ are the means of the weighted divergence sums for the AI-generated and real artwork distributions, respectively, and $\sigma_{𝒢}^{2}$ and $\sigma_{ℛ}^{2}$ are their variances.

The existence of such weights is guaranteed by the statistical distinctiveness of AI-generated artworks in at least some frequency bands, which is a consequence of the fundamental limitations of generative models in perfectly replicating the statistical properties of real artworks across all frequency scales simultaneously.

Furthermore, the dynamic frequency attention mechanism in Eq (12) provides an adaptive way to emphasize the most discriminative frequency bands:

$$\alpha_{k}=\frac{\exp\left(W_{a}^{T}\cdot {𝐳}_{k}+b_{a}+\sum_{j=1}^{K}\beta_{k,j}\cdot W_{a,j}^{T}\cdot {𝐳}_{j}\right)}{\sum_{i=1}^{K}\exp\left(W_{a}^{T}\cdot {𝐳}_{i}+b_{a}+\sum_{j=1}^{K}\beta_{i,j}\cdot W_{a,j}^{T}\cdot {𝐳}_{j}\right)}\cdot \;\left(1+\gamma_{1}\cdot \frac{\|{𝐳}_{k}{\|}_{2}^{2}}{\frac{1}{K}\sum_{j=1}^{K}\|{𝐳}_{j}{\|}_{2}^{2}}+\gamma_{2}\cdot \frac{\text{entropy}({𝐳}_{k})}{\max_{j}\text{entropy}({𝐳}_{j})}\right)\cdot \;\left(1+\gamma_{3}\cdot \frac{D_{KL}({𝐳}_{k}\|{\bar{𝐳}}_{ℛ,k})}{\frac{1}{K}\sum_{j=1}^{K}D_{KL}({𝐳}_{j}\|{\bar{𝐳}}_{ℛ,j})}+\gamma_{4}\cdot \frac{\exp\left(-\frac{(r_{k}-r_{\text{opt}}{)}^{2}}{2\sigma_{r}^{2}}\right)}{\frac{1}{K}\sum_{j=1}^{K}\exp\left(-\frac{(r_{j}-r_{\text{opt}}{)}^{2}}{2\sigma_{r}^{2}}\right)}\right)$$ (36)

The attention weights $\alpha_{k}$ naturally align with the optimal weights $\omega_{k}^{*}$ through the learning process, as they both emphasize the frequency bands with the highest discriminative power.

Thus, we have established the existence of a frequency band decomposition and corresponding feature mappings that satisfy the inequality stated in the theorem, completing the proof. □

**Corollary 1 (Structured locality of creation anomalies).** *For an AI-generated artwork* x~𝒢, *there exists a set of spatial regions* $\Omega_{x}\subset \{1,2,\ldots ,H\}\times \{1,2,\ldots ,W\}$, *satisfying the following conditions:*

$$\frac{1}{\left|\Omega_{x}\right|}\sum_{(i,j)\in \Omega_{x}}A_{\text{map}}(i,j)>\gamma \cdot \frac{1}{HW-|\Omega_{x}|}\sum_{(i,j)\notin \Omega_{x}}A_{\text{map}}(i,j)\;and\frac{\left|\Omega_{x}\right|}{HW}<\eta \;and\frac{1}{\left|\partial \Omega_{x}\right|}\sum_{(i,j)\in \partial \Omega_{x}}A_{map}(i,j)>\frac{1}{\left|\Omega_{x}⧵\partial \Omega_{x}\right|}\sum_{(i,j)\in \Omega_{x}⧵\partial \Omega_{x}}A_{map}(i,j)$$ (37)

*where* $\left|\Omega_{x}\right|$ *represents the region size,* γ>1 *is the contrast coefficient,* η∈(0,1) *is the area constraint,* $\partial \Omega_{x}$ *represents the region boundary, and* $A_{\text{map}}$ *is defined by Eq* (14).

*Proof*: Building upon Theorem 1, we know that AI-generated artworks exhibit distinctive statistical patterns in certain frequency bands. Now, we will show that these artifacts are not uniformly distributed across the image but rather concentrate in specific spatial regions.

Consider the anomaly map $A_{\text{map}}$ defined in Eq (14):

$$A_{\text{map}}(i,j)=\sum_{k=1}^{K}\alpha_{k}\cdot \frac{{\left\|\phi_{k}(F_{i,j})-\mu_{ℛ,k}\right\|}_{2}^{2}}{\sigma_{ℛ,k}^{2}+\epsilon }\cdot \left(1+\eta_{1}\cdot \exp\left(-\frac{\|[i,j]-[i_{c},j_{c}]{\|}_{2}^{2}}{2\sigma_{c}^{2}}\right)\right)\cdot \;\left(1+\eta_{2}\cdot \frac{D_{KL}(p_{\phi_{k}(F_{i,j})}\|p_{\phi_{k}(F_{ℛ})})}{\max_{i^{\prime },j^{\prime }}D_{KL}(p_{\phi_{k}(F_{i^{\prime },j^{\prime }})}\|p_{\phi_{k}(F_{ℛ})})}\right)\cdot \;\left(1+\eta_{3}\cdot \frac{{\text{var}}_{\left(i^{\prime },j^{\prime })\in 𝒩(i,j\right)}(\phi_{k}(F_{i^{\prime },j^{\prime }}))}{\frac{1}{HW}\sum_{i^{\prime }=1}^{H}\sum_{j^{\prime }=1}^{W}{\text{var}}_{\left(i^{\prime \prime },j^{\prime \prime })\in 𝒩(i^{\prime },j^{\prime }\right)}(\phi_{k}(F_{i^{\prime \prime },j^{\prime \prime }}))}\right)$$ (38)

The anomaly map quantifies the deviation of local frequency domain features from the reference distribution of real artworks at each spatial position (*i*,*j*) in the image.

For an AI-generated artwork x~𝒢, we define a threshold *τ* such that:

$$\Omega_{x}=\{(i,j)|A_{\text{map}}(i,j)>\tau \}$$ (39)

This defines a set of spatial locations where the anomaly score exceeds the threshold, indicating potential AI-generated artifacts.

First, we need to show that the anomaly scores within $\Omega_{x}$ are significantly higher than those outside $\Omega_{x}$. Let ${\bar{A}}_{\text{in}}$ and ${\bar{A}}_{\text{out}}$ be the average anomaly scores inside and outside $\Omega_{x}$, respectively:

$${\bar{A}}_{\text{in}}=\frac{1}{\left|\Omega_{x}\right|}\sum_{(i,j)\in \Omega_{x}}A_{\text{map}}(i,j)$$ (40)

$${\bar{A}}_{\text{out}}=\frac{1}{HW-|\Omega_{x}|}\sum_{(i,j)\notin \Omega_{x}}A_{\text{map}}(i,j)$$ (41)

By construction, we have $A_{\text{map}}(i,j)>\tau$ for all $(i,j)\in \Omega_{x}$ and $A_{\text{map}}(i,j)\leq \tau$ for all $(i,j)\notin \Omega_{x}$. Therefore:

$${\bar{A}}_{\text{in}}>\tau \geq {\bar{A}}_{\text{out}}$$ (42)

We need to establish that ${\bar{A}}_{\text{in}}>\gamma \cdot {\bar{A}}_{\text{out}}$ for some γ>1. The value of *γ* depends on the discriminative power of the feature mappings $\phi_{k}$ and the attention weights $\alpha_{k}$.

From Theorem 1, we know that AI-generated artworks exhibit significant deviations from real artworks in certain frequency bands. These deviations are not uniformly distributed across the image but tend to concentrate in specific regions due to:

1. Generative models often struggle with specific semantic elements (e.g., facial features, texture transitions, complex geometric structures). 2. The generation process typically operates at multiple scales, with artifacts more pronounced at certain scales. 3. Boundary regions between different semantic elements often exhibit higher anomaly rates due to the challenges in modeling spatially coherent transitions.

Therefore, there exists a threshold *τ* such that:

$${\bar{A}}_{\text{in}}>\gamma \cdot {\bar{A}}_{\text{out}}$$ (43)

for some γ>1, which establishes the first condition.

For the second condition, we need to show that $\frac{\left|\Omega_{x}\right|}{HW}<\eta$ for some η∈(0,1). This follows from the observation that AI artifacts are typically localized rather than pervasive throughout the image. If $\left|\Omega_{x}\right|$ were too large, it would contradict the premise that AI-generated artworks are capable of producing mostly realistic imagery with only specific regions exhibiting detectable anomalies.

The value of *η* can be determined empirically based on the capabilities of state-of-the-art generative models. As these models improve, we expect *η* to decrease, reflecting the increasing difficulty in distinguishing AI-generated from real artworks. However, due to fundamental limitations in perfectly modeling the statistical properties of real artwork distributions, *η* remains bounded away from zero.

Finally, for the third condition, we need to establish that the average anomaly score along the boundary $\partial \Omega_{x}$ is higher than in the interior $\Omega_{x}⧵\partial \Omega_{x}$:

$$\frac{1}{\left|\partial \Omega_{x}\right|}\sum_{(i,j)\in \partial \Omega_{x}}A_{\text{map}}(i,j)>\frac{1}{\left|\Omega_{x}⧵\partial \Omega_{x}\right|}\sum_{(i,j)\in \Omega_{x}⧵\partial \Omega_{x}}A_{\text{map}}(i,j)$$ (44)

The boundary $\partial \Omega_{x}$ is defined as:

$$\partial \Omega_{x}=\{(i,j)\in \Omega_{x}|\exists (i^{\prime },j^{\prime })\notin \Omega_{x}\text{ such that }\|(i,j)-(i^{\prime },j^{\prime }){\|}_{1}=1\}$$ (45)

This condition follows from the nature of generative artifacts at transition regions. AI generative models often struggle with maintaining consistency at the boundaries between different semantic elements or texture regions. These transition areas require modeling complex spatial dependencies that are particularly challenging for current generative architectures.

The local frequency analysis captured by:

$$\frac{{\text{var}}_{\left(i^{\prime },j^{\prime })\in 𝒩(i,j\right)}(\phi_{k}(F_{i^{\prime },j^{\prime }}))}{\frac{1}{HW}\sum_{i^{\prime }=1}^{H}\sum_{j^{\prime }=1}^{W}{\text{var}}_{\left(i^{\prime \prime },j^{\prime \prime })\in 𝒩(i^{\prime },j^{\prime }\right)}(\phi_{k}(F_{i^{\prime \prime },j^{\prime \prime }}))}$$ (46)

in Eq (14) is particularly sensitive to these boundary inconsistencies, resulting in higher anomaly scores along $\partial \Omega_{x}$.

Additionally, the structural property of generative models to handle different image regions somewhat independently (through mechanisms like attention or convolution) leads to statistical discontinuities at region boundaries that are captured by the cross-spectral analysis in Eq (11).

Therefore, all three conditions are satisfied, establishing the structured locality of creation anomalies in AI-generated artworks. This structured locality provides a powerful signature for distinguishing between real and AI-generated artworks, even as the quality of AI generation continues to improve. □
